# Caesarean Section on Maternal Request: An Italian Comparative Study on Patients’ Characteristics, Pregnancy Outcomes and Guidelines Overview

**DOI:** 10.3390/ijerph17134665

**Published:** 2020-06-29

**Authors:** Luisa Masciullo, Luciano Petruzziello, Giuseppina Perrone, Francesco Pecorini, Caterina Remiddi, Paola Galoppi, Roberto Brunelli

**Affiliations:** Department of Maternal and Child Health and Urological Sciences, Sapienza, University of Rome, Policlinico Umberto I, Viale del Policlinico 155, 00161 Rome, Italy; giuseppina.perrone@uniroma1.it (G.P.); francesco.pecorini@uniroma1.it (F.P.); remiddi.1451057@studenti.uniroma1.it (C.R.); paola.galoppi@uniroma1.it (P.G.); roberto.brunelli@uniroma1.it (R.B.)

**Keywords:** caesarian section on maternal request, caesarian section, pregnancy outcome

## Abstract

In recent years, the rate of caesarean sections has risen all over the world. Accordingly, efforts are being made worldwide to understand this trend and to counteract it effectively. Several factors have been identified as contributing to the selection of caesarean section (CS), especially an obstetricians’ beliefs, attitudes and clinical practices. However, relatively few studies have been conducted to understand the mechanisms involved, to explore influencing factors and to clearly define the risks associated with the caesarean section on maternal request (CSMR). This comparative study was conducted to elucidate the factors influencing the choice of CSMR, as well as to compare the associated risks of CSMR to CS for breech presentation among Italian women. From 2015 to 2018, a total of 2348 women gave birth by caesarean section, of which 8.60% (202 women) chose a CSMR. We found that high educational attainment, use of assisted reproductive technology, previous operative deliveries and miscarriages within the obstetric history could be positively correlated with the choice of CSMR in a statistically significant way. This trend was not confirmed when the population was stratified based on patients’ characteristics, obstetric complications and gestational age. Finally, no major complications were found in patients that underwent CSMR. We believe that it is essential to evaluate patients on a case-by-case basis. It is essential to understand the personal experience, to explain the knowledge available on the subject and to ensure a full understanding of the risks and benefits of the medical practice to guarantee the patients not only their best scientific preparation but also human understanding.

## 1. Introduction

Scientific progress, as well as social, cultural and legal changes, have led to a transition as far as caesarean section (CS) is concerned. Therefore, the consensus around the indications for CS has changed in many countries, now including psychosocial factors such as anxiety about the delivery, or even CS on maternal request (CSMR) in the absence of any medical indication [1]. Nevertheless, the reasons for increasingly liberal attitudes toward CS are different and not always easily discernable.

The rate of caesarean sections increased from 11% in 1980 to 30% in 2015, which is significantly above the World Health Organization (WHO) recommendations of 15–20% [2,3]. According to the latest global estimates, the average CS rate is approximately 25–30%, with large discrepancies between and within different countries [4]; in fact, while several African countries have a CS rate of 1–2% [4], between 25% of all deliveries in the United States and Canada are performed by CS, and in Latin America CS rates exceed 40%, reaching 80% in the private sector [4,5,6].

According to the last update of the Certificate of Delivery Care (CeDAP), in Italy, the CS rate is 33.7% [7]. This percentage makes Italy the European country with the highest rate of CS [7]; with a higher disproportion among regions (20% in Northern Italy versus 56% in Southern Italy), among private (51%) and public hospitals (29%). Changing risk profiles among increasingly older primiparae are often cited as a reason for the rise in caesarean deliveries [8,9,10,11]. Among these factors, a maternal request of caesarean sections plays an important role [11,12]; in fact, in the last years, Italian rate of CSMR increased significantly from 4.5% to 9% of all CS [13]. 

The American College of Obstetricians and Gynecologists (ACOG) define CSMR as an elective caesarean section in absence of a standard medical/obstetrical indication in order to avoid vaginal birth [14,15]. 

High CS rates are strongly influenced by financial, social and cultural factors [16,17,18,19,20,21]. These factors, especially combined with the public perception that a caesarean delivery is now an almost risk-free procedure, may well be contributing to the rise in the number of CS performed. 

Improvements in surgical technique and improvements in safety have led mothers to think of caesarean delivery as a viable alternative to vaginal delivery, even in the absence of any maternal or fetal indication [22,23,24]. This perspective, however, overlooks the fact that a CS is a surgical procedure with numerous potential complications for both mother and child. Pregnant women requesting a CS in the absence of obstetric indications has become a highly debated issue in academic as well as popular literature [25].

From a medical point of view, both maternal and neonatal morbidity is undoubtedly higher in abdominal births compared to vaginal ones. Despite there being a lower risk of hemorrhages (0.02% versus 0.07%) and chorioamnionitis (0.01% versus 0.08%) compared to vaginal birth, maternal morbidity due to infections (0.6% versus 0.2%), thromboembolic injuries (0.06% versus 0.03%) and anesthetic complications (0.5% versus 0.2%) [26] could be considered. In particular, the complications related to later pregnancies should be mentioned: uterine rupture [25,27], infertility [28,29,30,31], or even placental anomalies, such as placenta previa, increta, or accreta [1,9,31,32,33,34,35,36]. Uterine rupture is estimated worldwide to be at 1% and it is directly related to types of incision (from 0.7% to 9%) [25,26,27]. As regards infertility, some studies estimate a fertility reduction of 23.2% in women who have a second pregnancy [28,29,30,31]. Incidence of placental abnormalities are directly associated with the number of CS performed, with a percentage that increases from 1.3% to 3.3% [1,9,31,32,33,34,35,36].

Several studies have tried to understand the causes of CSMR comparing caesarean delivery to vaginal delivery [37,38,39,40]. Findings were often contradictory due to different patients’ characteristics, but obstetric providers were well-aware about risks and benefits of an elective and natural birth.

Therefore, it would be useful to analyze maternal and fetal outcomes, analyzing specific characteristics of two different populations: pregnant women that requested caesarean section and those who performed it for an obstetric indication. Investigating this theme could help health providers to improve clinical counselling.

The aim of our research is to identify demographic, clinical characteristics and obstetric outcomes of pregnant women that request a caesarean delivery and those who perform it for breech presentation.

International guidelines on CSMR are reviewed and discussed, making some practical proposals to reduce the rate of CSMR.

## 2. Materials and Methods

### 2.1. Study Population

From 2015 to 2018, we carried out a retrospective examination of hospital records. Among these, we compared patients who underwent CSMR and a control group who performed elective CS for breech presentations at Policlinico Umberto I in Rome.

The inclusion criteria were:

- Singleton pregnancies
- Italian pregnant women
- Elective caesarean sections performed for maternal request. This occurred only after extensive interviews with obstetricians, midwives and anesthetist of our department to explain the knowledge available on the subject and to understand risks and benefits of this medical practice.
- Maternal request of caesarean delivery during the latent or active phase of labor.
- Maternal refusal to undergo the induction of labor or subsequent failure to such induction and subsequent request of caesarean delivery.

The exclusion criteria were:

- Twin pregnancies
- Maternal comorbidity
- Presence of previous caesarean sections in their obstetric history
- Presence of maternal or fetal diseases that indicates Caesarean section.

The following characteristics were analyzed by reviewing patient medical records: maternal age, body mass index (BMI: weight/height^2^), education, employment, onset of pregnancy, parity, type of previous deliveries, previous abortions, gestational age of birth, obstetric complications of pregnancy and delivery, length of hospitalization, neonatal weight and APGAR index.

Information about maternal risk factors, comorbidity, obstetric complications, interventions during pregnancy and delivery were recorded by codes, according to International Classification of Diseases ninth revision (ICD–9). This enabled the exclusion of women with any preexisting medical risk factors, as well as children with any congenital malformations, to prevent possible confounders.

All women underwent monthly obstetric examinations and several ultrasound scans (US) that evaluated fetal presentation, biometrical measures and Doppler flowmetry, by following SIEOG (Società italiana di Ecografia Ostetrico Ginecologica) guidelines [41]. If there were no contraindications, all women who were near term, with breech presentations, were offered an external cephalic version attempt, under US guidance and cardiotocography (CTG) control. Unsuccessful external cephalic version confirmed fetal breech presentation which, according to Italian guidelines, represented an indication for elective caesarean section [42].

In general, the definitions of mode of delivery follow Robson classification [43], which uses obstetric characteristics to categorize all women admitted for delivery into one of ten groups. This classification represents the global standard for comparing and monitoring CS rates in hospitals. CSRM was not included in Robson classification, but our department allowed this procedure following these steps: women who requested CS underwent an extensive interview with obstetricians, midwives and anesthetists of our department to explain the knowledge available on the subject and to understand the risks and benefits of this medical practice; after that, all women signed a written informed consent confirming their choice.

### 2.2. Statistics

Statistical analysis was conducted using Real Statistics Resource Pack software (Release 6.8; copyright (2013–2020) Charles Zaiontz (www.real-statistics.com)). Descriptive analyses were presented as frequency with percentage, mean and standard deviation for all variables considered. Student T-Test for non-categorical variables and Chi-Framework Test for categorical variables were used to evaluate the significance; *p*-value <0.05 was considered as statistically significant. Multivariate logistic regression was performed with the following parameters: alpha 0.05, classification cut-off 0.5, iteration 20 and Newton’s method.

### 2.3. Ethical Approval

All patients provided written informed consent and procedures followed were in accordance with the Helsinki declaration of 1975, as revised in 2000.

The study protocol was approved by the scientific local department committee (Protocol number 002/2016).

## 3. Results

During the time period analyzed, 2348 caesarean sections were performed, of which 202 (8.60%) were CSMR and 209 (8.90%) were CS for breech presentation. In 2015 7.53% (*n* = 30) were CSMR and 8.07% (*n* = 65) in 2016. In 2017 they increased to 8.92% (*n* = 60); in 2018 CS represented 9.93% (*n* = 47). The percentage described above increased by 2.5% over the four years analyzed.

We analyzed that elective CS requested during pregnancy represented 55% of CSMR; those requested during latent phase of labor were 25%; 11% of patients requested CS as a refusal of any other method of induction and those who required it after a lack of induction response were 9%.

Main patients’ characteristics are reported in Table 1.

As far as maternal age is concerned, we observed a statistically significant increase in maternal age between those requesting CS and the control group, with a mean of 33.9 and 28.8, respectively (*p* = 0.002). In addition, there was a higher proportion of women older than 40 years among cases: 16.8% of women were aged 40–44 (compared to 0.5% in the control group) and 5.9% of women were aged over 45 years (compared to none in control group).

Regarding education, there was a statistically significant difference between the two groups (*p* < 0.001) In addition, we found that among CSMR the educational level was higher, with 36.6% of graduates, in comparison with 15.3% in the control group. These data could be related to employment; in fact, there was a statistically significant difference among employed (67.3% of cases versus 46.4% of controls) and unemployed patients (32.7% of cases versus 53.6% of controls, *p* = 0.004).

Considering the onset of pregnancy, it is interesting that although the majority of pregnancies were spontaneous, there was an increase of 15.4% in assisted reproductive technology (ART) amongst the group of patients that requested CS compared to the 1.9% in the control group (*p* < 0.001).

According to parity, there were 83.2% of primiparous women in the case group compared to 67.5% in the control group; multiparous women represented 16.8% of cases and 32.5% of controls (*p* = 0.035).

It was estimated that 14% of patients that requested CS had a previous operative delivery, performed using suction cup or forceps (*p* = 0.043).

Another factor to consider is the presence of previous miscarriages in the obstetric history: we found a statistically significant difference between the two groups, with 39.4% of the cases analyzed having voluntary termination of pregnancies (VTPs), as opposed to 18.7% in the controls (*p* = 0.032).

Obstetric morbidity and neonatal outcomes are described in Table 2.

Although the mean gestational age at birth was the same across the two groups (38 weeks), we found 13% of women that request CS had a gestational age older than 40 gestational weeks compared with only 2% in the control group. No statistically significant difference was found between these two groups.

The length of hospital stay was slightly longer in the group of CSMR (*p* = 0.004). No major complications were found; long-term hospitalization was due to minor complications.

Multivariate logistic regression analysis demonstrated a slight correlation between CSMR and the variables analyzed.

We calculated incidences and relative risks (RR) for every variable and we could conclude that, considering each feature individually, CSRM was chosen by a woman’s specific phenotype. In particular, patients older than 35 years had a higher risk of CSMR if compared to other age groups (from a minimum RR = 1.19 compared to 30–34 years old to a maximum RR = 3.35 compared to 20 years old).

The university level of education was statistically significant toward the CSMR, in comparison to patients with high school education (RR = 1.634), with middle school degree (RR = 1.727) and with elementary school education (RR = 1.677).

Higher BMI (>35) also showed statistically significant results for the choice of CSMR in comparison to a lower BMI (<24) with RR = 1.227.

Moreover, patients who underwent ART procedures were more prone to choose a CSMR compared to women with a spontaneous onset of pregnancy (RR = 1.949). The same result involved the deliveries >40 weeks of gestation in comparison to deliveries between 38 and 39 weeks (RR = 1.89) to deliveries <37 weeks (RR = 1.696).

The multiparous women showed, on the contrary, a lower probability of CSMR in comparison to primiparous patients (RR = 0.613). Finally, no other significant correlation was observed.

## 4. Discussion

In Italy, the CS rate is 33.7% [7]—far above the limits set by the WHO recommendations [3]. Among several factors, an increment in maternal request of caesarean sections (CSRM) might have contributed to the increase of CS [11,12].

This study aimed to investigate the frequency and clinical profile of patients that request caesarean deliveries on maternal request compared to patients who underwent planned caesarean sections for obstetric indication, specifically evaluating maternal and neonatal outcomes. We have reached this goal because maternal request has been recorded for research in the medical charts of our University hospital.

Several studies have compared CSMR to vaginal delivery and analyzed groups’ characteristics, obstetric outcomes, short-term complications in puerperium and showed no increase in major complications in CS [39].

ACOG’s committee has approved CSMR with some restrictions: it can only be performed after 39 weeks’ gestation (in order to allow fetal lung maturation) and it should not be performed in women who desire multiple pregnancies (due to the increase risks of placenta accreta and previa). Furthermore, CSMR should not be motivated by ineffective pain management [15].

In this study, only 55% of CSMR sample choose an elective caesarean delivery, the remaining half of the sample self-determinate at a time immediately before the delivery or at the time of pharmacological induction.

Moreover, it was found that a high percentage of patients over 40 years old with a better socio-economic and cultural profile are among those women that request caesarean sections. These findings corroborate international literature [44,45].

As far as the patients’ obstetric history is concerned, we observed a high percentage of women who achieved pregnancy using ART. In general, this percentage is also likely to be underestimated because patients who undergo medically assisted procreation methods frequently tend to turn to private facilities. It would be interesting to understand the reasons why these women choose CS: this choice could be influenced by previous miscarriages in their obstetric history or the desire to protect such a precious pregnancy. Therefore, these women could choose this mode of delivery as they consider it safer for themselves but especially for their child [44,45]. It is important to observe the high percentage of patients that experienced an operative delivery and VTPs or therapeutic miscarriages among cases: these women, in fact, shared a traumatic experience that could have influenced their future choices [18,46].

Furthermore, our results show no significant differences in major complications of CSMR in comparison to elective CS for obstetric indication.

A possible explanation of a longer hospitalization among cases could be attributed to the treatment of minor complications such as fever, pain relief and surgical suture infections.

Notably, a recent Danish study from 2019 showed no increase in major complications associated with CSMR but an increased rate of wound infections [39]. This research identifies the women who ask for CS and confirms the exclusion of major complications in women that request caesarean delivery, as previously mentioned. These data are reassuring but leave a problem unsolved: how obstetricians should behave following the request for caesarean section.

However, increased reliability of caesarean birth and decreased rates of vaginal birth after CS play important roles in the current increased CS rates. An increasing number of CS increases the adhesion rate, which can cause additional increased morbidity directly or with peripheral organ injury. Abnormal placenta development following repeated caesarean birth is concurrent with an increased risk of placenta previa and abruptio placenta in addition to placenta accreta. The risk of placenta previa has been reported to increase by 0.28–2% in patients who have undergone at least one CS in a meta-analysis including 36 studies [18]. Hysterectomy is another significant morbidity. It is mostly associated with placenta accreta, placenta previa, uterine atony and uterine rupture. Each uterine scar is accompanied with an increasing risk of hysterectomy, independent of the presence of placenta previa [47].

Clinicians should take into consideration two things: primarily, international guidelines do not give a strict consensus but place emphasis on the physicians personal choice; secondly, there is a concurrent need to decrease number of CS within the limits set by WHO. According to investigations carried out in recent years, two of the most important elements of the high rate of CS in Italy could be the repeated CS and CSMR.

Concerning the ethics of the CSMR, the debate focuses on the motivations of providers and patients. It is the obstetricians’ imperative to evaluate the balance between risks (especially long-term risks) and benefits and try to understand the reasons behind this decision [48]. Consequently, CSMR remains a highly controversial topic and is still well-debated.

### International Guidelines on CSMR

International organizations have long discussed the acceptability of this medical procedure. In 2002, the International Federation of Gynecology and Obstetrics (FIGO) reported that performing a caesarean section without medical indications is to be considered ethically unjustified. Recently there was a change in the attitudes of national and international organizations on this issue, causing a considerable increase in the demand for CSMRs. The FIGO guidelines seemed to be more open-minded than others about this mode of birth.

Unfortunately, the current status quo is that there are not enough data [49] to provide any concrete practical recommendation related to CSMR. A comprehensive evaluation of various results suggests that although there are no important differences between an elective caesarean section performed by maternal choice and an expected vaginal delivery, the evidence is too weak to definitively conclude that there are no differences between the two modes of birth. In the absence of well-designed randomized controlled clinical trials, there is no scientific evidence about the real risk–benefit ratio of the CSMR [50]. Meanwhile, various obstetric societies have attempted to explain this phenomenon through the best available evidence.

The SOGC (Society of Obstetricians and Gynecologists of Canada) guidelines suggest that caesarean sections should not be performed in the absence of medical or obstetric indications, being reserved only for obstetric conditions with a real threat to the health of the mother or fetus [51]. Clinical and ethical issues of caesarean section on maternal request were also addressed by the (ACOG): according to these guidelines, the attention of health personnel should be placed on the reasons behind the maternal request. The goal must be to provide unbiased information regarding the benefits and risks of the procedure. In this way, women could express an autonomous and informed choice. [15] Elective caesarean section should only be performed after the 39th week of gestation, to reduce the risk of neonatal respiratory morbidity. Moreover, the mother’s wish to have more children should also be evaluated in order to avoid unwanted complications in subsequent pregnancies. [52]

The Royal Australian and New Zealand College of Obstetricians and Gynecologists (RANZCOG) [53] states that if after a thorough and complete discussion the patient requests a caesarean section as a delivery mode, the treating doctor could:

(1) accept to perform the caesarean section after having verified that the woman has fully understood the risks of the procedure she intends to undergo;
(2) refuse to perform the caesarean section if they believe that there will be significant risks to maternal or neonatal health or if the patient has not adequately understood the possible complications of the procedure;
(3) advise the patient to seek the opinion of another professional.

The document of the National Institute for Health and Care Excellence (NICE) [54] on caesarean section believes that an appropriate approach for women who require a CSMR is to explore, research and discuss the reasons behind the request, thus individualizing the management. The need to provide information is highlighted especially through taking into consideration women’s concerns and priorities. Informed consent should be signed only after verifying that such information, presented in an accessible and understandable way, has been adequately understood.

Since the most common reason for requesting a caesarean section is tocophobia, an adequate assessment of women’s fears and counseling could lead to, at least, half of women choosing a vaginal birth [55] and being satisfied with their choice.

If, despite several counselling sessions, the woman continues to request a caesarean delivery and she rejects the idea of a vaginal birth, a caesarean section should be offered for the overall benefit of the mother and child. It is important to ensure that women have accurate understanding that the partner, and, if necessary, the family are involved in the decision-making process so that the woman has all the support she needs.

Finally, the Italian guidelines of Istituto Superiore di Sanità (ISS) on caesarean section [42] agree that the best way to approach the phenomenon of maternal self-determination is by an in-depth interview between the doctor and the patient. Obstetricians should illustrate the available evidence on the subject and they should evaluate the reasons that lead the woman to make this request. Specific individual meetings and information support represent some of the most important steps for a patient’s decision. These interventions have proven to be useful in reducing the levels of anxiety and fear related to the birth event and in improving the subjective experience of childbirth. In the absence of an appropriate medical indication to perform a caesarean section, the doctor has the right to refuse the request of the patient, guaranteeing the woman the possibility of accessing a second clinical opinion.

Main guideline positions about CSMR are summarized in Table 3.

## 5. Conclusions

This study describes the main characteristics and outcomes of a sample of Italian women who ask for CS and confirm the exclusion of the major complications related to this procedure. According to these results, the strategies aimed to reduce the CSMR must consider other topics and not exclusively the surgical risks.

Although CSMR is still a little investigated concept, we believe it is essential to evaluate patients on a case-by-case basis. It is important to establish a good relationship with the patients, in order to deepen the motivations of their choice. It will be necessary to understand the personal experience, to explain the knowledge available on the subject and to ensure a full understanding of the risks and benefits, in particular the long-term reproductive complications.

It is important that the partner is involved in the decision-making process, and, if necessary, the family, so that they have all the support they need. The CSMR is a highly multifactorial dimension based on personal experiences, individual characteristics (advanced maternal age, ART onset of pregnancies, negative obstetric history, external conditioning, particular attitude of the patient towards the present pregnancy) and many other factors that should be well understood by obstetricians. Clinicians must commit every day to guarantee the patient not only his best scientific preparation but also human understanding: in fact, among the health providers involved, obstetricians are the most supportive of a woman’s decision [56].

In our opinion, since there is not yet a complete agreement on the CSMR, if a woman, after an extensive counseling, persists in that request, she should receive a CS to preserve her from a negative psychological experience.

Further studies could help to demonstrate the efficacy of an intervention strategy including a convinced obstetric support together with psychological aid.

## Figures and Tables

**Table 1:** Patients’ characteristics.

Demographic and Clinical Characteristics	Caesarean Section for Maternal Request (*n* = 202)		Caesarean Section for Breech Presentation (*n* = 209)		*p*-Value
**Age (m ± SD)**	33.9 ± 6.2		28.8 ± 6.4		0.002
	*n*	%	*n*	%	
<20	4	2.0%	15	7.2%	
20–24	11	5.4%	34	16.2%	
25–29	29	14.4%	78	37.3%	
30–34	58	28.7%	40	19.1%	
>35	100	49.5%	42	20.2%	
**Education**					<0.001
Elementary school	5	2.5%	7	3.3%	
Middle school	40	19.8%	59	28.2%	
High school	83	41.1%	111	53.1%	
University	74	36.6%	32	15.3%	
**Employment**					0.004
Employed	136	67.3%	97	46.4%	
Unemployed	66	32.7%	112	53.6%	
**BMI (m ± SD)**	28.5 ± 4.9		28.09 ± 5.3		0.521
<18.5	0	0%	1	0.6%	
18.5–24.99	44	21.7%	57	27.2%	
25–29.99	85	42%	80	38.7%	
30–34.99	50	24.8%	51	24.5%	
>35	23	11.5%	20	9.6%	
**Onset of pregnancy**					<0.001
Spontaneous	171	84.6%	205	98.1%	
ART	31	15.4%	4	1.9%	
**Parity**					0.035
Primiparous women	168	83.2%	141	67.5%	
Multiparous women	34	16.8%	68	32.5%	
**Previous mode of delivery**					0.043
Vaginal delivery	37	86.0%	90	100.0%	
Operative delivery (suction cup/forceps)	6	14.0%	-	0.0%	
**Previous Miscarriages**					0.032
Spontaneous miscarriages	66	60.6%	65	81.3%	
VTPs	43	39.4%	15	18.7%	
**Gestational age (m ± SD)**	37 w 5 d ± 2 w 3 d		37 w 6 d ± 1 w 5 d		0.151
<37 weeks	42	21%	42	20%	
38–39 weeks	132	66%	162	78%	
>40 weeks	28	13%	5	2%	

m: mean; SD: standard deviation; ART: assisted reproductive technology; VTP: voluntary termination of pregnancy; w: weeks; d: days.

**Table 2:** Obstetric morbidity and neonatal outcomes.

Obstetric Morbidity and Neonatal Outcomes	Caesarean Section for Maternal Request (*n* = 202)		Caesarean Section for Breech Presentation (*n* = 209)		*p*-Value
**Obstetric complications**	*n*	%	*n*	%	0.0731
Hypertension	28	22.4%	9	13.4%	
Gestational diabetes	18	14.4%	13	19.4%	
Oligohydramnios/Polyhydramnios	28	22.4%	16	23.9%	
PROM	26	20.8%	25	37.3%	
IUGR	25	20.0%	4	6.0%	
Postpartum hemorrhage	3	1.5%	2	1.3%	
**Hospitalization (m ± SD)**	3.6 d ± 2.6		2.4 d ± 1.6		0.004
1–3 days	126	62.4%	189	90.4%	
4–6 days	50	24.7%	12	5.7%	
7–9 days	17	8.4%	5	2.4%	
>9 days	9	4.5%	3	1.5%	
**Neonatal weight (m ± SD)**	3055 g ± 651		3105 g ± 675		0.2923
≤1500 g	3	1.5%	1	0.5%	
≤2500 g	23	11.4%	28	13.4%	
2500–4000 g	172	85.1%	178	85.2%	
>4000 g	4	2%	2	0.9%	
**APGAR 1 (m ± SD)**	8.40 ± 0.92		8.53 ± 0.95		0.14222
**APGAR 5 (m ± SD)**	9.50 ± 0.80		9.60 ± 0.89		0.1533

PROM: premature rupture of membrane; IUGR: intrauterine growth restriction; d: days; g: grams

**Table 3:** International guideline overview.

Guidelines	Year	Position about CSMR
**FIGO** [49]	2002	Opposed but open-minded to certain conditions
**ACOG** [15]	2007	Open-minded, evaluating risks and benefits
**SOGC** [51]	2009	Opposed
**NICE** [54]	2011	In favor, after extended counselling sessions and informed consent
**RANZCOG** [53]	2013	In favor, after extended counselling sessions and informed consent
**ISS** [42]	2014	In favor, after extended counselling sessions and informed consent
**ACOG** [52]	2019	More open-minded, evaluating risks and benefits
